# A Deep Reinforcement Learning and Graph Convolution Approach to On-Street Parking Search Navigation

**DOI:** 10.3390/s25082389

**Published:** 2025-04-09

**Authors:** Xiaohang Zhao, Yangzhi Yan

**Affiliations:** 1 School of Civil Engineering, Dalian University of Technology, Dalian 116024, China; 2 Department of Architecture and Civil Engineering, School of Engineering, City University of Hong Kong, Hong Kong 999077, China; yangzhi@seu.edu.cn; 3 China-Pakistan Belt and Road Joint Laboratory on Smart Disaster Prevention of Major Infrastructures, Southeast University, Nanjing 211189, China

**Keywords:** multi-agent reinforcement learning, graph neural networks, dynamic parking allocation, spatiotemporal optimization

## Abstract

**Highlights:**

**What are the main findings?**

- A Multi-Agent Reinforcement Learning (MARL) framework is proposed for dynamic parking allocation, integrating Graph Neural Networks (GNNs) to model spatial correlations and Gated Recurrent Units (GRU) to capture temporal variations in parking demand.
- Experimental results reveal that the proposed model greatly outperforms established methods (FIFO, SIRO) in optimizing parking resource allocation, lowering cruising time, and boosting parking success rates.

**What is the implication of the main finding?**

- The proposed framework provides a scalable and adaptive solution for real-time parking allocation, effectively addressing the challenges of spatiotemporal demand variability and enhancing urban parking management efficiency.
- By incorporating contrastive learning (CL) and heterogeneous graph pooling (HGP) to optimize spatial interaction modeling, the model enables more efficient decision-making in complex and dynamic parking environments.

**Abstract:**

Efficient parking distribution is crucial for urban traffic management; nevertheless, variable demand and spatial disparities raise considerable obstacles. Current research emphasizes local optimization but neglects the fundamental challenges of real-time parking allocation, resulting in inefficiencies within intricate metropolitan settings. This research delineates two key issues: (1) A dynamic imbalance between supply and demand, characterized by considerable fluctuations in parking demand over time and across different locations, rendering static allocation solutions inefficient; (2) spatial resource optimization, aimed at maximizing the efficiency of limited parking spots to improve overall system performance and user satisfaction. We present a Multi-Agent Reinforcement Learning (MARL) framework that incorporates adaptive optimization and intelligent collaboration for dynamic parking allocation to tackle these difficulties. A reinforcement learning-driven temporal decision mechanism modifies parking assignments according to real-time data, whilst a Graph Neural Network (GNN)-based spatial model elucidates inter-parking relationships to enhance allocation efficiency. Experiments utilizing actual parking data from Melbourne illustrate that Multi-Agent Reinforcement Learning (MARL) substantially surpasses conventional methods (FIFO, SIRO) in managing demand variability and optimizing resource distribution. A thorough quantitative investigation confirms the strength and flexibility of the suggested method in various urban contexts.

## 1. Introduction

The optimization of on-street parking allocation has become a critical issue in the face of accelerating urbanization. As parking demand continues to rise, particularly during peak hours, the limited availability of parking spaces exacerbates traffic congestion, increases carbon emissions, and significantly hinders overall urban mobility efficiency [1]. This issue not only contributes to immediate inefficiencies but also affects broader social and economic outcomes, such as increased travel time, reduced productivity, and higher operational costs for businesses and individuals. Studies emphasize that the spatial and temporal imbalance in parking demand is a key factor driving these inefficiencies in parking resource utilization [2]. High demand areas, such as central business districts, frequently face parking shortages, while peripheral regions remain underutilized due to a lack of effective allocation systems [3]. Addressing this imbalance is essential, not only to alleviate congestion and optimize parking in high-demand areas, but also to enhance overall urban efficiency, and to support sustainable, low-carbon transportation.

Various optimization solutions have been suggested to improve parking resource use and mitigate traffic inefficiencies, including dynamic parking assignment, intelligent guiding systems, and model-based allocation frameworks [4]. These systems automatically modify parking spot distribution based on real-time demand, hence minimizing search durations and parking failure rates. Recent research has integrated multi-agent systems (MAS) and reinforcement learning (RL) to enhance adaptive scheduling in intricate contexts [5,6]. Moreover, intelligent parking guidance systems employing sensors and vehicle-to-infrastructure (V2I) technologies have been deployed in many cities to aid drivers in finding available parking spaces, thereby mitigating congestion resulting from ineffective parking searches [7,8].

However, current approaches still encounter major difficulties when applied to the complexity of urban environments, even with the increased focus on dynamic parking distribution. Traditional static allocation methods, like first-in-first-out (FIFO) [9] and service-in-random-order (SIRO) [10], are insufficient for managing the extremely changing characteristics of parking demand. These methods fail to adapt to real-time variations, resulting in systemic inefficiencies and the suboptimal use of parking resources. Although more sophisticated approaches, such as online parking assignment systems [11], seek to address these difficulties, they frequently depend on unrealistic assumptions. This encompasses the constant accessibility of high-resolution, real-time data regarding parking requests and space occupancy, alongside centralized control systems capable of executing globally best decisions. In reality, these requirements are seldom fulfilled, especially during times of maximum congestion or unforeseen disruptions, where parking behaviors become erratic and the necessity for flexible, decentralized management is imperative.

Furthermore, the ongoing spatial disparity between parking availability and demand intensifies system inefficiencies. Areas with high demand frequently face significant shortages, whereas periphery locations are underutilized despite possessing adequate parking places. Despite recent research proposing dynamic reallocation strategies to tackle this issue, several analyses continue to neglect critical behavioral elements, such as parking costs, which profoundly affect user choices and demand distribution [12,13]. The model provided in this study does not consider pricing, which constitutes a shortcoming of the current research. Subsequent research will focus on incorporating pricing mechanisms into the optimization framework.

These constraints underscore the pressing necessity for intelligent, decentralized, and adaptive optimization frameworks that can respond to dynamic, partially observable environments [14]. Contemporary deep reinforcement learning models, despite their potential, frequently depend on centralized control frameworks, exhibit inadequate coordination among agents [11,15], and neglect to account for long-term effects such as parking time. These constraints impede their scaling and result in inferior performance [16]. This paper tackles these problems by presenting a multi-agent reinforcement learning system aimed at surmounting these limitations and providing a decentralized, scalable, and adaptable solution for real-time parking distribution.

To address these issues, this study proposes a multi-agent reinforcement learning framework tailored to the complex nature of dynamic on-street parking allocation. On-street parking is chosen as the focus of this study due to its significant impact on urban mobility and its unique characteristics, including high variability in demand across different times and locations, and its sensitivity to external factors such as traffic congestion and local events. The proposed framework is designed to be decentralized, scalable, and adaptive to real-time conditions, offering the following key contributions:

1. An MARL parking allocation framework is established to address the intricate and fluctuating characteristics of urban parking demand. The system facilitates cooperative decision-making among several actors to enhance real-time parking distribution. A DRL-based modeling approach is provided to improve the utilization and allocation efficiency of parking resources;
2. An innovative exploration technique is developed and included into the MARL framework to reduce the likelihood of premature random actions resulting in unsatisfactory long-term allocation strategies. The technique successfully captures spatiotemporal fluctuations in parking demand, ensuring quicker convergence and enhanced adaptation to intricate urban parking environments;
3. To substantiate the proposed framework, two prevalent baseline models, First In, First Out (FIFO) [9] and Service In Random Order (SIRO) [10] are employed for comparative analysis. A realistic parking simulation environment is created to enable quantitative performance analysis of various allocation algorithms. Experimental findings indicate that the proposed MARL framework markedly surpasses conventional baselines regarding parking resource efficiency, demand equilibrium, and overall allocation efficacy.

The subsequent sections of this work are structured as follows: Section 2 examines the current literature regarding parking allocation. Section 3 delineates the parking allocation problem and presents baseline methodologies for comparative evaluation. Section 4 delineates the proposed MARL framework, encompassing the DRL model, learning algorithms, and exploration methodologies. Section 5 delineates the experimental configuration and simulation outcomes, offering a comparative assessment of various allocation methodologies. Section 6 ultimately encapsulates the principal contributions of this study and delineates prospective avenues for further investigation.

## 2. Related Work

### 2.1. Methods for Allocation and Optimization of Parking Spaces

Reservation-based parking techniques enable drivers to prebook parking spots, hence minimizing the time spent searching for a parking spot upon arrival at their location. Tan et al. [17] employed game theory to analyze the interplay between reservation-based and search-based parking tactics, concluding that reservation-based techniques can markedly decrease drivers’ cruising time and overall travel expenses. Macea et al. [18] introduced a reservation-based behavioral model for managing parking demand in urban environments, taking into account drivers’ preferences for parking selection and duration to enhance the allocation of parking spaces and pricing techniques. Lu and Liao [19] created an intelligent parking reservation system employing deep learning methods to forecast parking availability and offer tailored parking suggestions based on drivers’ preferences.

On-street parking, especially in central metropolitan locales, poses a significant issue in traffic management owing to its fluctuating demand and the irregular spatiotemporal allocation of parking spots. Certain research has commenced by examining the dynamic distribution of on-street parking resources. Zhao et al. [20] introduced a demand-sensitive on-street parking guidance system named D2Park, which addresses the specific parking requirements of individual vehicles and offers customized parking suggestions based on their preferences to minimize cruising duration and walking distance. Zhang, Zhao, Liao, Li and Du [11] proposed an online parking allocation method for partially connected vehicles utilizing multi-agent deep reinforcement learning, which dynamically assigns parking spaces based on real-time data while accounting for the unpredictability of parking demand and availability. Awaisi et al. [21] devised a smart parking assistance system utilizing deep reinforcement learning, which analyzes previous data to offer drivers real-time parking suggestions, thereby minimizing search duration and enhancing parking spot efficiency.

In addition to reservation-based and search-based strategies, researchers have proposed other parking allocation strategies. Ahmed et al. [22] presented a blockchain-based architecture for integrated smart parking systems, aiming to address trust and performance issues in multi-party data sharing and provide a one-stop parking information service for commuters in smart cities, along with a set of design principles to demonstrate the system’s applicability. Furthermore, some studies have considered parking management strategies for specific vehicle types. Bischoff et al. [23] used intelligent agent technology to investigate parking management strategies for city-wide shared taxis. Jemmali et al. [24] proposed a parking space allocation framework based on the fair distribution of total people in each parking area, and developed a set of seven algorithms to reduce the gap in the number of people between parking areas, conducting extensive experiments on 2430 different cases and demonstrating superior performance compared to the best algorithms in the existing literature.

### 2.2. Optimization Methods for Dynamic Allocation Problems

Traditionally, classical dynamic resource allocation problems have depended on mathematical programming and heuristic techniques. Topaloglu and Powell [25] introduced an approximate dynamic programming method utilizing value function approximations for stochastic, multi-period problems. Spivey and Powell [26] devised an adaptive framework that amalgamates linear programming with optimality requirements for dynamic strategy modifications. Despite being beneficial in many instances, real-time resolution continues to pose challenges for large-scale, complicated decision-making problems.

Heuristic algorithms like the tabu search for dynamic vehicle routing proposed by Lai et al. [27], the multi-scenario sampling with re-optimization by Bent and Van Hentenryck [28], and the ant colony optimization tailored for dynamic routing by Montemanni et al. [29] have shown flexibility and effectiveness in real-time applications. To handle the increasing complexity, there is a trend towards hybrid algorithms. Chen and Xu [30] combined column generation with genetic algorithms for large-scale vehicle dispatch, and Ghiani et al. [31] created an adaptive search heuristic for vehicle routing.

Notwithstanding advancements, the unpredictability of dynamic situations and the necessity for real-time processing present considerable hurdles. The characteristics of reinforcement learning models and their online learning capabilities provide unique solutions for dynamic resource allocation, aiming for optimal strategies in changing settings.

### 2.3. Reinforcement Learning for Dynamic Optimisation

Conventional dynamic optimization techniques, including mathematical programming and heuristic algorithms, have exhibited exceptional efficacy under particular circumstances. Nonetheless, their real-time problem-solving capacities, adaptability, and generalization skills are constrained when confronted with extensive decision-making challenges. In recent years, reinforcement learning (RL) has emerged as a viable methodology, demonstrating distinct advantages in dynamic optimization challenges owing to its adaptability and generalization capacities. Mao et al. [32] effectively utilized deep Q-networks (DQN) for resource allocation issues, whereas Li et al. [33] introduced a policy gradient-based approach for vehicle routing optimization. These investigations have convincingly illustrated the formidable capacity of reinforcement learning in managing high-dimensional state spaces and dynamic situations.

Despite the numerous highlights of RL in the dynamic optimization domain, its practical application still faces several challenges. For instance, Dulac-Arnold et al. [34] pointed out that the training stability of RL needs improvement when dealing with complex constraints and sparse rewards. Furthermore, Rajeswaran et al. [35] discussed the sim-to-real gap, which refers to the differences between RL policies in simulation and real-world applications, placing higher demands on the robustness of the policies. Simultaneously, Doshi-Velez and Kim [36] emphasized the need to enhance the interpretability and trustworthiness of RL in practical applications, including policy visualization and constraint satisfaction proofs.

To address these challenges, some researchers have attempted novel approaches. Nagabandi et al. [37] used a combination of model predictive control (MPC) and RL to improve sample efficiency. Tobin et al. [38] developed domain randomization techniques to reduce the gap between simulation and reality. Gilpin et al. [39] enhanced the interpretability of RL by applying explainable machine learning methods.

Moreover, effectively balancing exploration and exploitation [40], designing appropriate reward functions [41], and solving multi-objective optimization problems are crucial in the dynamic optimization process [42]. Future research needs to achieve more breakthroughs in these areas to promote the application of RL in dynamic optimization problems, and provide more powerful and reliable tools for solving practical issues.

In summary, previous research has provided significant insights into parking allocation using reservation systems, heuristic algorithms, and reinforcement learning methodologies. Nevertheless, the majority of these studies depend on centralized decision-making, static optimization models, or constrained spatial–temporal representations, rendering them less effective in rapidly changing metropolitan settings. This study presents a decentralized multi-agent reinforcement learning system that incorporates graph convolutional networks to model spatial dependencies and gated recurrent units to capture temporal dynamics. This integration facilitates adaptive, scalable, and real-time parking allocation, overcoming the constraints of current methodologies regarding coordination, flexibility, and responsiveness amid varying demand situations.

## 3. Problem Statements

### 3.1. Challenges in the Allocation of Dynamic Parking Spaces

Urban parking demand has considerable spatiotemporal variability, shaped by elements like historical parking data, real-time traffic patterns, road network configuration, the distribution of parking spaces, and sites of interest (POIs). Empirical research demonstrates that in high-demand regions, especially within central business districts (CBDs), drivers often expend 5 to 15 min seeking an accessible parking space, with parking-related cruising contributing to over 30% of overall urban traffic congestion. Extended parking search durations result in heightened car emissions and fuel usage, while also intensifying road network inefficiencies, thereby diminishing overall urban mobility. Conversely, low-demand locations frequently exhibit the underutilization of parking spaces, leading to considerable spatial disparities in resource distribution [3]. Traditional parking allocation methods, including first-in-first-out (FIFO) and random allocation (SIRO), exhibit insufficient adaptation to dynamic demand variations, resulting in inferior system efficacy. Moreover, in areas of high demand, parking failure rates persistently remain elevated, as the scarcity of available spaces compels drivers to prolong their search or engage in illicit parking, hence intensifying urban congestion and amplifying the incidence of parking offenses.

Confronting these difficulties requires a sophisticated, data-informed optimization strategy that utilizes real-time data to adaptively modify parking resource distribution. Nevertheless, current static parking management methods predominantly depend on historical data and do not integrate real-time occupancy information, therefore proving inadequate in addressing short-term demand fluctuations. The progression of intelligent parking technologies, such as parking meter transaction data and integrated parking sensors, offers novel options to improve the real-time decision-making efficacy of parking allocation systems. Parking sensors, placed at specific parking spaces, consistently monitor occupancy status and relay high-frequency updates, facilitating accurate assessment of real-time parking availability. Simultaneously, parking meters enhance this data by documenting transaction timestamps, thus aiding in the assessment of parking turnover rates and duration distributions [8]. Integrating real-time data sources with historical occupancy trends enables the development of predictive models that can forecast short-term variations in parking demand, thereby establishing a solid basis for improving allocation tactics.

**Figure 1:**
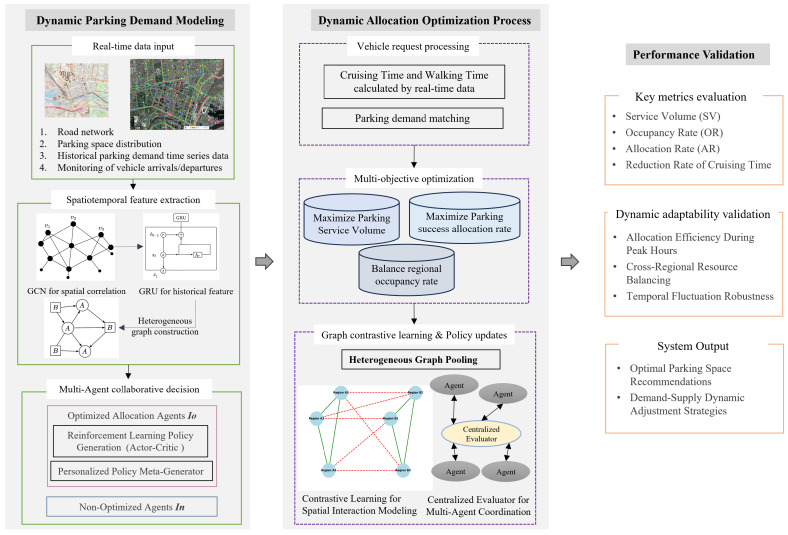
Overview of method framework.

This study utilizes a graph convolutional network (GCN) to represent spatial connections among parking locations and gated recurrent units (GRU) to analyze temporal demand changes, illustrated in Figure 1, therefore efficiently capturing the intricate spatiotemporal patterns of parking demand. The capacity to derive significant representations from sensor data allows the system to adapt parking allocations dynamically according to real-time conditions. This study proposes a scalable, intelligent parking management framework that integrates real-time occupancy data with machine learning predictive modeling to tackle ongoing issues of parking supply–demand discrepancies, prolonged cruising durations, and suboptimal resource allocation. The incorporation of parking sensor data and transaction records into the decision-making process signifies a crucial advancement in creating an adaptable, real-time parking management system that improves urban mobility and mitigates congestion-related inefficiencies.

### 3.2. Problem Formulation for Dynamic Parking Allocation

**Definition** **1** **(Parking request).** *A parking request $q_{t}=<l_{t},T_{t}>\in Q\;$ is defined as the t-th request generated by the parking platform within a single day, corresponding to the t-th time step. In this context, $l_{t}\;$ denotes the location at which the vehicle initiates the request, and $T_{t}\;$ indicates the time of initiation. The completion time $T_{t}^{\prime }\;$ is determined dynamically by the system environment based on the outcome of the parking process. The set of all parking requests is denoted as $Q=\left\{q_{1},\;q_{2},\ldots ,q_{t}\right\}$, which, combined with the available on-street parking resources, constitutes a complex and dynamically evolving urban parking system.*

**Definition** **2** **(Total travel time).** *The total travel time for a user is defined as the sum of cruising time and walking time. Cruising time ${\;\eta }_{p}^{t}\;$ refers to the time a vehicle spends searching for an available parking space in roadside parking lot p, starting from the moment the parking request is submitted through the platform. This request is assumed to be initiated when the vehicle has already arrived in the vicinity of its intended destination, represented by the corresponding spatial grid within the city.*

The model defines the proximity to the destination based on the distance or travel time between the vehicle’s current grid and the candidate parking locations. According to the simulation settings, the search radius is constrained to a range between 0 and 20 km. Therefore, being in the vicinity of the destination implies that the vehicle is located within an adjacent spatial area and has begun the active process of searching for parking.

The cruising time is calculated as follows:

(1) $$\eta_{p}^{t}=e_{p}{\left(1-\frac{\delta_{p}^{t}}{\sigma_{p}}\right)}^{-1},\;\forall t,p,$$

where $e_{p}$ represents the average cruising time for roadside parking lot p when it is vacant; $\delta_{p}^{t}$ and $\sigma_{p}$ represent the current occupancy rate and the maximum capacity of parking lot p, respectively. The walking time from parking lot p to destination i is denoted as $W_{pi}$. The total travel time is thus given by

(2) $$Total\;travel\;time=\eta_{p}^{t}+W_{pi}\;\;$$

**Definition** **3** **(Parking service volume).** *The parking service volume of a parking operator $P_{k}$ is defined as the total number of parking requests served by its parking spaces. This metric is a key indicator for evaluating the utilization rate of the parking facilities operated by $P_{k}$.*

**Definition** **4** **(Parking allocation success rate).** *The parking allocation success rate is defined as the proportion of successfully fulfilled parking requests to the total number of requests received within a given area $P_{k}$. This metric reflects the effectiveness of the allocation strategy in matching supply with demand over time.*

At each time step throughout the day, the parking platform continuously receives a sequence of parking requests $q_{t}\in Q$. The objective of this task is to learn an optimal allocation policy that determines, for each incoming request $q_{t}$, whether to accept it and assign it to one of the parking facilities managed by the operator $P_{k}$, or to reject it when no suitable space is available. The long-term goal is to maximize the number of successfully completed parking requests, improve the overall allocation success rate, and minimize total travel time. Unlike greedy strategies such as FIFO, the policy must dynamically balance spatiotemporal demand distribution, resource availability, and future system states to achieve both system-wide efficiency and fairness.

This task is formulated as a Markov Decision Process (MDP). At each time step, the agent observes the current system state and decides whether to accept and allocate the parking request. The objective is to learn a policy that maximizes the expected cumulative reward, which incorporates allocation success rate, travel time, and system efficiency. The environment embeds key constraints, including parking capacity, parking space assignment, and user waiting time limits. The detailed reward design is provided in Section 4.2.5.

## 4. Methodology

### 4.1. Overall Framework of Reinforcement Learning

The proposed reinforcement learning framework aims to dynamically optimize urban parking resource allocation through a structured multi-agent approach. The process begins with state representation, where real-time and historical data are integrated to derive key environmental variables, including current occupancy levels, forecasted parking demand, active vehicle parking requests, and the availability of parking spaces. To effectively capture the spatiotemporal dependencies inherent in parking systems, Graph Convolutional Networks (GCNs) are employed to model spatial correlations across parking zones, while Gated Recurrent Units (GRUs) are utilized to capture temporal dynamics. These extracted features constitute a high dimensional observation space that serves as the input for intelligent decision-making agents.

**Figure 2:**
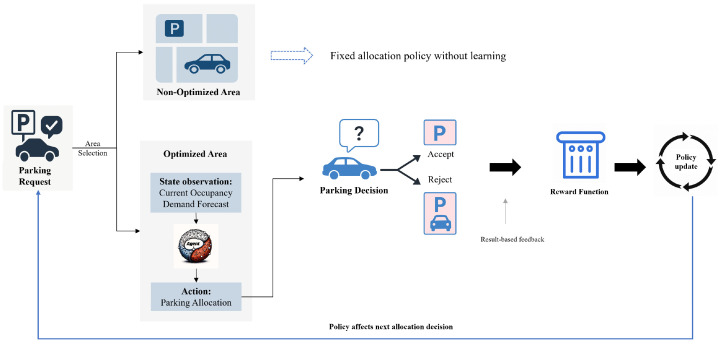
Framework of reinforcement learning model.

Based on the observed states, agents generate actions that determine parking space allocations. The action space consists of two types of agents—(1) optimized agents, which leverage reinforcement learning to adaptively adjust their allocation strategies, and (2) static agents, which operate with fixed rule-based policies in non-learning regions. This hybrid agent structure allows the system to maintain decision-making stability in non-optimized areas while promoting adaptive behavior in critical regions. The overall interaction process is illustrated in Figure 2, which outlines parking requests, processed through observation, decision-making, feedback, and iterative policy updates.

To guide the learning process, a delayed reward function is designed to balance multiple objectives; it penalizes excessive cruising time and incentivizes successful parking allocations. When a parking request is successfully completed, a reward is returned based on system-level efficiency and demand–response considerations. This reward mechanism enables agents to learn long-term optimal strategies through experience.

The policy learning process is further enhanced through multi-agent coordination using the multi-agent deterministic policy gradient framework. In this setup, a centralized evaluator supports joint learning by sharing information across agents, which improves the overall allocation efficiency. Through continuous interactions between state observation, decision-making, reward feedback, and policy updates, the framework enables real-time, scalable, and adaptive parking management in dynamic urban environments.

### 4.2. State, Action, Reward

#### 4.2.1. Agent

The set of agents is defined as $I=[I_{o},I_{n}]$, where each roadside parking facility $c_{i}\in I$ is regarded as an individual agent. These agents are categorized into two distinct types: optimized allocation agents $I_{o}$, which dynamically adjust parking allocation strategies based on real-time parking demand and supply–demand equilibrium, and non-optimized allocation agents $I_{n}$, which adhere to pre-established parking management policies without engaging in the optimization process. The optimized allocation agents, $I_{o},$ leverage adaptive decision-making mechanisms to enhance the efficiency of parking resource utilization, whereas the non-optimized allocation agents, $I_{n}$, operate under static allocation rules.

#### 4.2.2. State

The observation space is defined as $O=O_{1}\times \cdots \times O_{N}$, with the corresponding state space denoted as S. At the t-th parking request $q_{t}$, the observation of agent $c_{i}$, represented as $o_{i}^{t}\in O_{i}$ consists of two main components—agent-specific attributes and information provided by the parking platform. The agent-specific attributes include a unique identifier for each agent, its affiliated parking management entities, and the number of available parking spaces at its location. The parking platform provides real-time information, including the time step $T_{t}$ at which the parking request occurs, the geographical distance from the request location $l_{t}$ to the parking facility $c_{i}$, the estimated time of arrival, and the number of vehicles currently cruising toward $c_{i}$ in search of parking. Additionally, the observation incorporates predicted future parking demand in the vicinity of $c_{i}$, which is estimated based on historical data and predictive models. The joint observation at the *t*-th step, which consists of the observations of all agents, is denoted as $O_{t}$ and formally expressed as $O_{t}=[o_{1}^{t},o_{2}^{t},\cdots ,o_{N}^{t}]$. The system state at time step t is represented as $S_{t}\in S$, which aggregates the observations of all agents, reflecting the overall distribution of parking resources and serving as the foundation for parking resource optimization and allocation decisions.

#### 4.2.3. Action

The action space is defined as $A=A_{1}\times \cdots \times A_{N}$. At time step t the action of agent $c_{i}$, denoted as $a_{i}^{t}\in A_{i}$, represents its service decision in response to the parking request $q_{t}$. For optimized allocation agents $c_{i}\in I_{o}$, actions are dynamically generated in real time based on a reinforcement learning model, allowing the system to adapt to real-time parking demand and supply–demand equilibrium. In contrast, non-optimized allocation agents $c_{i}\in I_{o}$ operate under predefined management strategies, where their actions are determined in advance and remain fixed regardless of individual parking requests $q_{t}$. The joint action at time step t, representing the collective decisions of all agents, is formally expressed as

(3) $$A_{t}=[a_{1}^{t},a_{2}^{t},\cdots ,a_{N}^{t}]\in A,$$

This joint action integrates the dynamically optimized decisions of reinforcement learning-based agents with the static operational strategies of fixed-rule agents, serving as the basis for implementing parking resource allocation policies within the management system.

#### 4.2.4. State Transition

At time step t, the state transition of the environment is governed by the transition function $T=\left(S_{t+1}|S_{t},A_{t}\right)$, where T:S×A→S defines the probabilistic mapping from the current state $S_{t}$ to the subsequent state $S_{t+1}$ under the influence of the joint action $A_{t}$. The evolution of $S_{t+1}$ is influenced not only by the newly generated parking request $q_{t+1}$ but also by a combination of environmental factors, including variations in parking resource availability, the dynamics of cruising vehicles, and the cumulative impact of historical decision-making. Specifically, following the processing of the parking request $q_{t}$, at time step t, the system updates supply–demand conditions based on the current state $S_{t}$ and joint action $A_{t}$. As a new parking request $q_{t+1}$ emerges, the system further refines the allocation of parking resources, resulting in the transition to the next system state $S_{t+1}$. This transition process encapsulates the interplay between real-time parking demand, dynamic supply adjustments, and strategic decision-making, forming the foundation for the optimization of parking resource allocation.

#### 4.2.5. Reward Function

A delayed reward design is proposed, where the reward function $r\left(S_{t},A_{t}\right):S\times A\to R$ is returned only after the completion of parking request $q_{t}$ at time step t. The reward is defined as

(4) $$r_{t}=\{\begin{array}{cc} \varepsilon_{a}-\lambda cruising\;time, & success\;inI_{o}\\ \varepsilon_{a}, & failure\;in\;I_{o}\\ 0, & otherwise \end{array},$$

where $\varepsilon_{a}$ is a base incentive to encourage requests to be directed to optimized agents in $I_{o}$. λcruising time penalizes prolonged cruising, promoting faster and more efficient allocation behavior. While the numerical value of the reward may appear smaller in successful cases due to the subtraction, it represents the net benefit of an effective allocation and is always higher than the reward for failure or inaction. The “otherwise” case refers to parking requests that are either routed to non-optimized areas, $I_{n}$, or rejected before reaching an actionable decision point, due to lacking availability or timeout. In such cases, no reward is returned to the agent.

In the multi-agent reinforcement learning framework, the rewards are shared among all $I_{o}$ agents in the optimized allocation zone, facilitating cooperative decision-making. In contrast, for $I_{n}$ agents in non-optimized allocation zones, whose policies remain fixed, rewards are not considered. The optimized agents $I_{o}$ compete with $I_{n}$, while also cooperating to maximize the expected cumulative reward across all parking requests Q throughout the day,

(5) $$R_{t}=\sum_{t^{\prime }=t}^{\left|Q\right|}\gamma^{\left(t^{\prime }-t\right)}r_{t^{\prime }},$$

where γ∈[0,1] represents the discount factor. The comprehensive roadside parking system is administered by the Melbourne government, with parking spots distributed according to district divisions. The core region is designated for optimum allocation, whereas surrounding areas adhere to established distribution statutes.

Furthermore, to improve system performance and account for real-world limitations, a maximum user waiting time is established. If a parking request is not completed within the specified time frame, it is deemed unsuccessful, and no reward will be granted. This promotes the acquisition of more effective and prompt allocation tactics.

### 4.3. Adaptive Interaction Modelling for Dynamic Parking Allocation

In dynamic parking allocation, parking facilities consistently engage within a competitive and changing market landscape. The accurate modeling of inter-agent interactions is crucial, as a facility’s capacity to attract vehicles relies not only on its own characteristics, such as pricing and availability, but also on its relationships with adjacent facilities, encompassing both competition and collaboration. An accurate depiction of these interactions improves situational awareness and enables efficient parking allocation decisions. Nonetheless, two principal obstacles must be confronted when simulating these interactions at an urban scale.

The initial problem is defining the extent of interactions. In extensive urban areas, the quantity of parking facilities is considerable, rendering it computationally impractical and unwarranted to represent all paired interactions. Not all facilities exert a substantial influence on each other, especially when they are geographically remote. This study organizes parking facilities into regional partitions according to their adjacency relationships. Interactions are mostly analyzed inside and across spatially proximate areas, where facilities either directly compete for demand or collaborate to optimize parking availability. This systematic method guarantees that the interaction network encompasses the most pertinent competitive and cooperative factors while preserving computing efficiency.

The second challenge involves comprehending the nature of inter-agent interactions. Parking decisions are affected by various factors, such as occupancy rates, proximity to destinations, and walking distances. Competitive interactions occur when neighboring establishments cater to overlapping demand, prompting consumers to select between them based on immediate availability. Conversely, collaborative partnerships arise when many facilities jointly manage surplus demand, like when overflow from a completely occupied venue is channeled to proximate alternatives. These interactions are influenced by spatial proximity and temporal variations in demand, requiring a modeling technique that accurately reflects their changing attributes.

This study utilizes a graph-based interaction modeling framework that integrates a Graph Attention Network to tackle these difficulties. The parking system is depicted as a dynamic heterogeneous graph, with nodes representing parking facilities and edges indicating adjacency-based interactions. A two-stage graph attention mechanism is presented to adaptively weight the influence of surrounding facilities, rather than utilizing static aggregation strategies. At each time step, a facility dynamically consolidates information from its neighbors, prioritizing the most pertinent competitive influences, such as proximate facilities with analogous demand patterns, while diminishing the weight attributed to less significant interactions. This approach facilitates a context-sensitive depiction of parking market dynamics, guaranteeing the system’s responsiveness to variations in spatial and temporal settings.

The preference for the Graph Attention Network over conventional static aggregation techniques is driven by its capacity to dynamically learn and modify interaction weights. In the graph attention network, each parking facility node dynamically assesses the significance of its neighbors according to their conditions. When adjacent parking zones display comparable geographical attributes and temporal demand trends, the model allocates more attention weights to their interactions. In contrast, neighbors with diminished spatial or temporal relevance are assigned lesser weights. This selective aggregation allows the model to prioritize more significant interactions in the decision-making process.

All attention weights are normalized via a Softmax function, guaranteeing that the aggregate of weights for neighboring nodes equals one. This architecture improves the clarity and reliability of information synthesis.

### 4.4. Adaptive Meta Learning for Strategy Generation

In extensive dynamic parking allocation, training separate policies for each parking request would result in significant computational burden, rendering real-time decision-making impractical. This study employs an adaptive meta-policy learning framework to enhance computational efficiency and improve policy adaptability by utilizing a shared policy network to produce region-aware personalized policies. The proposed method utilizes a meta-policy generator alongside a Gated Recurrent Unit (GRU) to extract long-term parking features, rather than training distinct policies for individual agents, and employs a learnable hypernetwork to dynamically modify agent-specific policy parameters. The GRU is tasked with documenting historical parking characteristics, encompassing occupancy trends, variations caused by holidays and weather conditions, and discrepancies in parking failure rates. These temporal attributes function as the meta-representation of each agent, and are articulated as

(6) $$H_{t}^{i}=GRU(H_{t-1}^{i},[x_{t}^{i}∥a_{t-1}^{i}]),$$

where $H_{t-1}^{i}$ is the GRU output from the previous time step, $x_{t}^{i}$ is the current input feature derived from the reinforcement learning model, and $a_{t-1}^{i}$ is the allocation action from the previous step. This formulation enables agents to retain a memory of parking demand patterns across different regions, ensuring that policies are adapted to long-term variations in parking demand. Based on the extracted meta-features $H_{t}^{i}$, a learnable hypernetwork G is introduced to generate agent-specific parameters for personalized allocation strategies,

(7) $$W_{G,t}^{i}=G_{w}(H_{t}^{i}),b_{G,t}^{i}=G_{b}(H_{t}^{i}),$$

These parameters define the individualized decision-making strategies for each agent, ensuring that allocation policies are continuously adjusted according to historical occupancy patterns and environmental fluctuations. Each agent $c_{i}\epsilon I_{o}$ determines its allocation action using a personalized policy network, formulated as

(8) $$a_{t}^{i}=u_{t}^{i}(x_{t}^{i})=Sigmoid(W_{G,t}^{i}\phi (x_{t}^{i})+b_{G,t}^{i})\times \kappa ,$$

where $\phi (x_{t}^{i})$ represents a learnable transformation function, such as a neural network, responsible for mapping input features to a latent representation, while κ serves as a scaling factor that adjusts the output to align with the practical priority range for parking allocation. To address the challenge of strategy generalization across heterogeneous parking environments, the proposed framework incorporates a region-specific adaptation mechanism that mitigates the potential drawback of policy homogenization in shared-policy architectures. First, the GRU captures regional demand variations, ensuring that learned strategies account for long-term differences between urban centers and residential areas. Second, the policy adaptation mechanism is updated through an online reinforcement learning process that allows policies to dynamically optimize over time, preventing performance degradation due to shifting demand conditions. Finally, the hypernetwork dynamically generates agent-specific policy parameters, ensuring that the shared framework retains the flexibility required for individual decision-making.

### 4.5. Graph-Based Representation Learning for Large-Scale Parking Allocation

In the context of multi-agent reinforcement learning (MARL), Lowe et al. [43] proposed a decentralized execution and centralized training framework, where the observations and actions of all agents (i.e., $O_{t}$ and $A_{t}$) are used as inputs for the state and joint action in order to facilitate the training process. However, in the context of our roadside parking allocation problem, directly using the joint observation $O_{t}$ and joint action $A_{t}$ presents two main issues.

Firstly, it fails to sufficiently model the intricate interactions among agents, which are critical for accurately capturing the systemic dynamics and emergent trends within the parking market. Secondly, as the number of agents increases, the dimensionality of the joint observations and actions escalates, leading to substantial scalability issues, most notably the curse of dimensionality, in large-scale multi-agent systems such as the one considered in this study.

To address these issues, we propose a pooling representation module, designed to learn a compact yet semantically rich global representation of the parking market. This representation serves as an effective state-action representation, supporting centralized learning for large-scale agent systems. For simplicity, in this section, we omit the time step subscript t.

#### 4.5.1. Multi-View Heterogeneous Graph

Building on the theoretical framework of modeling the entire roadside parking market as a “dynamic heterogeneous graph”, we introduce a multi-view heterogeneous graph method designed to extract high-dimensional data from the parking market and distil them into a more compact and informative representation. Specifically, we begin by integrating the actions of dynamic parking allocation agents $I_{O}$ into the graph G by concatenating the actions with their respective observations. This process aggregates the parking allocation information for all agents, thus constructing a complete representation of the parking market, denoted as $G^{c}$. We then apply the model to $G^{c}$ in order to capture the interactions among all agents, resulting in enhanced observations for each agent, expressed as

(9) $$X=W_{p}∙G^{c},$$

where $W_{p}$ represents a learnable parameter, and X encompasses the enhanced observations for both dynamic and fixed agents, denoted as $\left\{X_{O,\;}\;X_{N}\right\}$, which are essential for modeling the complex interdependencies within the parking market. Subsequently, a learnable pooling operation is performed to extract key features from the parking market, generating a latent representation while eliminating redundant information. In particular, the projection vector $p_{O}$ is used to map $X_{O}$ into importance scores for the agents,

(10) $$Y_{O}=X_{o}p_{O},$$

where $p_{O}$ is a learnable projection vector. Based on the learned importance scores $Y_{O}$, we select the top $k_{h}$ most significant agents and discard the others, as follows:

(11) $$X_{O}^{topk},\;Y_{O}^{topk}=Filter\;(X_{o},\;Y_{o},\;k_{h}),$$

Here, $Y_{O}^{topk}$ represents the importance scores of the top $k_{h}$ agents. A gating operation is then employed to control the retention of relevant information. The importance scores are normalized into a gating vector, which is element-wise multiplied with $X_{O}^{topk}$,

(12) $$X_{O}^{gate}=X_{O}^{topk}⊙Norm(Y_{O}^{topk}),$$

In this context, ⊙ denotes element-wise multiplication, and the normalization function Norm is implemented via the Softmax operation. This gating mechanism allows gradients to propagate through the projection vector $p_{O}$, facilitating its learning through backpropagation. By learning {$X_{O}^{1,\;gate},\;\cdots ,X_{O}^{N_{topk},\;gate}$}, a permutation-invariant readout operation is applied to extract a comprehensive representation from the dynamic parking allocation agents,

(13) $$s_{O}=\sum_{i=1}^{N_{topk}}x_{O}^{i,gate}⊘{max}_{i=1}^{N_{topk}}x_{o}^{i,gate},$$

Similarly, a comprehensive representation $s_{F}$ is derived for the fixed parking allocation agents $I_{N}$, using distinct learnable projection vectors and the remaining number of agents. Ultimately, the latent representation of the entire parking market is expressed as

(14) $$H=[s_{O}∥s_{F}],$$

In summary, the proposed framework combines the competitive and cooperative dynamics of the parking market, providing a comprehensive solution to the issues of large-scale agent coordination and interaction.

#### 4.5.2. Contrastive Graph Representation Learning for Multi-Agent Coordination

A direct challenge concerns how to effectively train the Heterogeneous Graph Pooling (HGP) method to extract meaningful latent representations of the roadside parking market. One straightforward approach is to update the HGP through the reinforcement learning (RL) objective function. However, RL algorithms optimize agent policies based on feedback rewards from the environment, making this approach inherently more complex compared to supervised learning methods. Consequently, learning effective latent representations from high-dimensional input through RL is a challenging task. Inspired by contrastive learning in image-based models, we propose a Graph Contrastive Learning (GCL) objective as an auxiliary task to promote the learning of latent state-action representations from the high-dimensional roadside parking market.

Specifically, given a query instance $H_{q}$, a positive instance $H_{+}$, and K−1 negative instances $H_{\_}=\{H_{\_}^{1},\cdots ,H_{\_}^{K-1}\}$, the contrastive learning objective is designed to encourage the query instance $H_{q}$ to align with the positive instance $H_{+}$, while pushing it away from the negative instances $H_{i}^{\_}\in H_{\_}$ in order to learn distinguishable representations. The query instance $H_{q}$ is derived by applying HGP on the subgraph $G_{q}\in G^{c}$, as follows:

(15) $$H_{q}=HGP(G_{q}),$$

The position $l_{t}$ of the parking request $q_{t}$ is selected as the center, and the $k_{c}$ nearest parking facilities, along with their corresponding edges, are chosen to form the subgraph $G_{q}$. This process is analogous to cropping a subregion from a geographical map. Similarly, the subgraph for the positive instance $G_{+}$ is extracted from the same graph $G^{c}$, but with a randomly selected center. The subgraphs for the negative instances $G_{\_}^{i}$ are randomly cropped from other graphs within the batch. The representations for both the positive instance $H_{+}$ and the negative instances $H_{\_}$ are obtained using the same method.

The loss function is then employed to optimize the GCL objective function,

(16) $$L_{c}=-log\frac{exp(H_{q}^{Τ}W_{c}H_{+})}{\exp\left(H_{q}^{Τ}W_{c}H_{+}\right)+\sum_{i=1}^{K-1}\exp\left(H_{q}^{Τ}W_{c}H_{i}^{-}\right)},$$

A bilinear projection $W_{c}$ is used to assess the similarity between instances. $W_{c}$ is a learnable parameter. The objective function $L_{c}$ is treated as an auxiliary task and is optimized alongside the RL objective.

### 4.6. Optimized Centralized Policy Learning

A centralized actor–critic policy learning framework is proposed to improve the efficiency of multi-agent decision-making in extensive, dynamic parking allocation. This method is especially applicable in scenarios where agents need to continuously modify parking availability and pricing, providing a more refined control compared to discrete-action models. The centralized training and decentralized execution model facilitates global coordination while ensuring computational practicality in large agent networks. The actor–critic architecture stabilizes policy updates by utilizing both individual agent policies and a common evaluator, effectively tackling the non-stationarity challenges present in decentralized learning systems.

At each time step, given the joint observation and the dynamic heterogeneous graph representation of the system, the policy module and the meta-generator module are updated to maximize the estimated expected return,

(17) $$J(\theta_{G},\theta_{M})=E_{O_{t},G_{t}∽D}[Q_{u}(H_{t})|a_{i}^{t}=u_{i}^{t}(x_{i}^{t})],$$

where $\theta_{G}$ and $\theta_{M}$ are the learnable parameters of the model and meta-generator, respectively, and D represents the experience replay buffer containing state-transition tuples $\left(O_{t},A_{t},G_{t},O_{t+1},G_{t+1},r_{t}\right)$. The latent representation $H_{t}$ is computed at time step t, and $Q_{u}$ serves as the centralized evaluator shared across all agents, used to estimate the expected return.

To mitigate the non-stationarity issues inherent in decentralized multi-agent learning, a centralized evaluator aggregates global information while maintaining computational efficiency.

The optimization of both the centralized evaluator and the contrastive heterogeneous graph pooling learning module is achieved by jointly minimizing the reinforcement learning objective and the graph contrastive learning objective, formulated as

(18) $$L(\theta_{Q},\theta_{p})=E_{O_{t},A_{t},G_{t},O_{t+1},G_{t+1},r_{t}∽D}[{\left(Q_{u}(H_{t})-y_{t}\right)}^{2}]+\lambda L_{c},$$

where $y_{t}=r_{t}+\gamma Q_{u}^{\prime }(H_{t+1})|a_{i}^{t+1}=u_{i}^{t+1}(x_{i}^{t+1})$, and $\theta_{Q}$ and $\theta_{p}$ are the learnable parameters of the evaluator $Q_{u}$ In this context, $u_{i}^{t+1}$ and $Q_{u}^{\prime }$ denote the target policy of agent $c_{i}$ and the target evaluator for delayed parameters, respectively.

To further enhance policy learning robustness, graph contrastive learning is incorporated as an auxiliary objective to refine latent state action representations. By maximizing the similarity between augmented positive representations while increasing the divergence from negative representations, the proposed framework ensures more informative feature encoding.

## 5. Experiment

### 5.1. Data Description

This study uses real-world on-street parking sensor data collected in Melbourne in 2019 to support parking resource optimization [44]. The dataset includes the number of parking slots, travel distances and durations between parking facilities and demand grids, as well as time-series data on parking demand and supply. Each parking slot is linked to corresponding parking meter and sensor records, enabling the accurate tracking of usage behaviors. The data cover nine districts: Carlton, Docklands, East Melbourne, Kensington, Melbourne CBD, North Melbourne, Parkville, Southbank, and West Melbourne, comprising a total of 541 parking slots.

**Figure 3:**
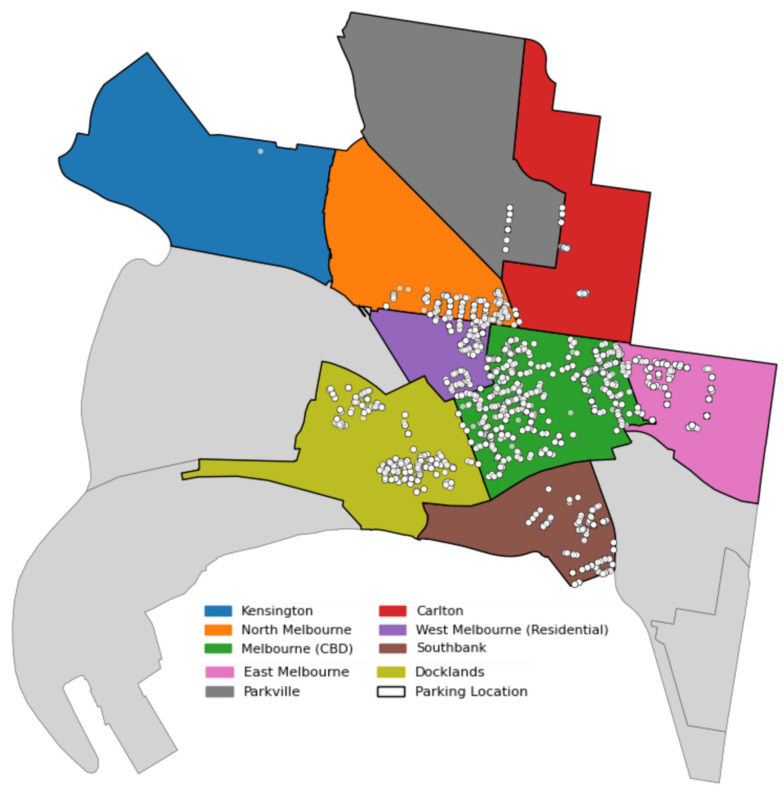
Parking locations and study areas across Melbourne.

The experimental period spans from 27 March to 11 May 2019, covering 46 days. During this period, an average of 29,342 parking requests were recorded per day across all regions. Detailed statistics on the number of parking slots and the corresponding daily parking requests per district are summarized in Table 1. Figure 3 illustrates the spatial layout of these nine key areas and the distribution of parking locations based on the geo-tagged sensor records. This spatial mapping provides essential insights into the urban structure and serves as a foundation for modeling spatiotemporal allocation dynamics.

**Figure 4:**
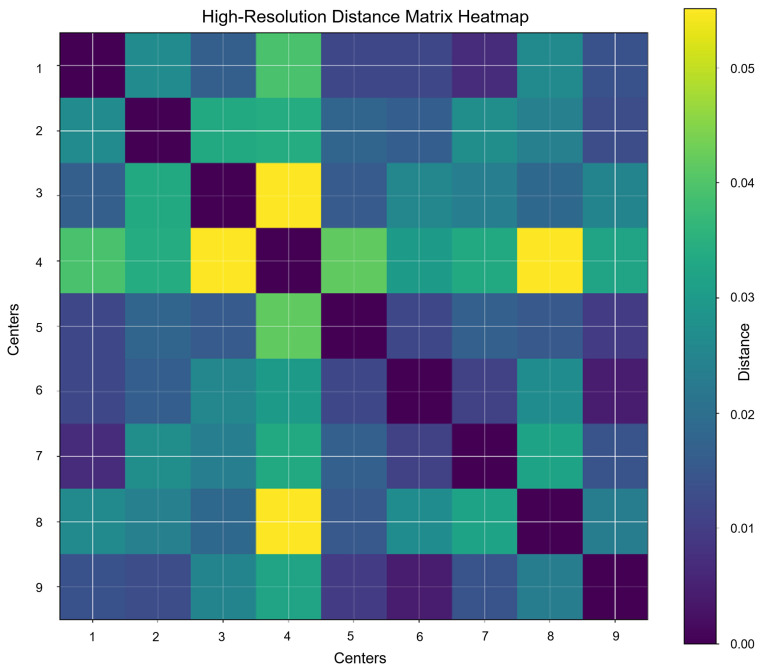
Normalized distance matrix between parking centers.

To further analyze the spatial relationships between parking facilities and demand grids, a high-resolution distance matrix heatmap was constructed, as shown in Figure 4. This heatmap visualizes the relative proximity between parking centers, where color intensity indicates normalized distance. Parking duration data and normalized distance matrices were used to capture the spatial structure of the system. All distance values were scaled to the range of 0 and 1 to eliminate unit inconsistencies and ensure comparable interpretation across regions. These preprocessing steps collectively enable the precise modeling of parking behaviors and support the subsequent reinforcement learning framework.

### 5.2. Model Training

A multi-agent reinforcement learning (MARL) framework was used to optimize parking resource allocation. The dataset was divided into training and validation sets, with approximately 80% for training and 20% for validation. To ensure consistency, demand and supply data were normalized across regions and time periods, and segmented into discrete time steps to capture temporal dynamics. During training, a single day was randomly sampled in each iteration. The validation set was used only for testing the learned policy. Moreover, two baseline strategies were adopted for comparison, as follows:

- **First-in first-out (FIFO)** [9]. Allocates parking spaces in the order requests arrive, without considering demand variation or spatial context;
- **Service In Random Order (SIRO)** [10]. Assigns spaces randomly, lacking coordination or learning capabilities.

Policy learning was guided by a discount factor of 0.99 and adaptive learning rate scheduling. MARL agents learn through interaction and localized observations, enabling coordinated and adaptive allocation strategies. Model performance was evaluated using the following metrics:

- **Service Volume (SV)**—The total number of parking requests that were successfully served by the system during the simulation period;
- **Occupancy Rate (OR)**—The proportion of parking spaces utilized;
  (19) $$OR=\frac{1}{T}\sum_{t}\frac{Number\;of\;occupied\;slots}{Total\;slots}$$
- **Allocation Rate (AR)**—The proportion of requests successfully assigned to parking slots
  (20) $$AR=\frac{Total\;successful\;requests}{Total\;requests\;submitted}$$

It is important to note that a high AR may coincide with a relatively low OR when parking durations are short or requests are distributed unevenly across time. In such cases, frequent turnovers lead to many fulfilled requests without sustained occupancy, highlighting the temporal dynamics of parking behavior rather than inefficiency in the allocation mechanism.

### 5.3. Model Performance

#### 5.3.1. Model Performance Comparison with Baseline

Table 2 compares the performance of the proposed MARL-GCN model with two baseline strategies, FIFO and SIRO. The MARL-GCN model achieves a service volume of 27,071, an occupancy rate of 66.42%, and an allocation rate of 92.24%, indicating strong allocation capability and efficient space utilization.

In comparison, the FIFO records a service volume of 19,309, an occupancy rate of 65.54%, and an allocation rate of 65.81%. The SIRO achieves a service volume of 20,945, an occupancy rate of 57.77%, and an allocation rate of 71.40%.

Moreover, according to Table 2, we completed the model training on a standard device equipped with an RTX 3060 GPU (16 GB VRAM) and an i7 CPU. During the inference phase, the model required only about 0.17 s to process a single parking request, which fully meets the real-time response requirements of urban-scale parking navigation systems.

Compared with rule-based methods such as FIFO and SIRO, our model introduces moderate computational overhead but achieves an approximately 20% improvement in allocation success rate. This demonstrates a well-balanced trade-off between resource allocation efficiency and computational speed, making it highly suitable for practical deployment.

#### 5.3.2. Model Performance in Different Zones

An analysis of model performance across different areas in Melbourne reveals notable regional disparities. As shown in Table 3, Melbourne CBD records the highest service volume at 14,918 and the highest allocation rate at 92.45%, reflecting intense parking demand and strong model responsiveness. In contrast, Docklands and Parkville show lower service volumes of 1523 and 417, respectively, along with relatively low allocation rates of 81.57% and 84.80%, indicating sparse demand and limited agent activity in low-density areas.

Kensington, with only one daily parking request, contributes marginally to system performance. Mid-demand zones such as North Melbourne and Carlton achieve high allocation rates of 91.20% and 90.30%, suggesting stable model behavior under moderate demand conditions. Occupancy rates range from 61.29% in Docklands to 68.02% in the CBD, highlighting spatial heterogeneity in utilization intensity.

**Figure 5:**
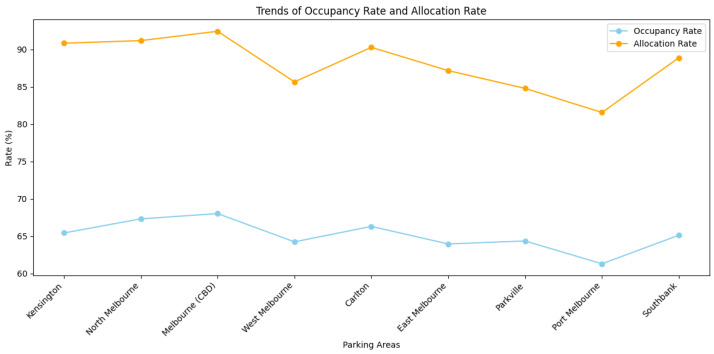
Trends of occupancy rate and allocation rate.

**Figure 6:**
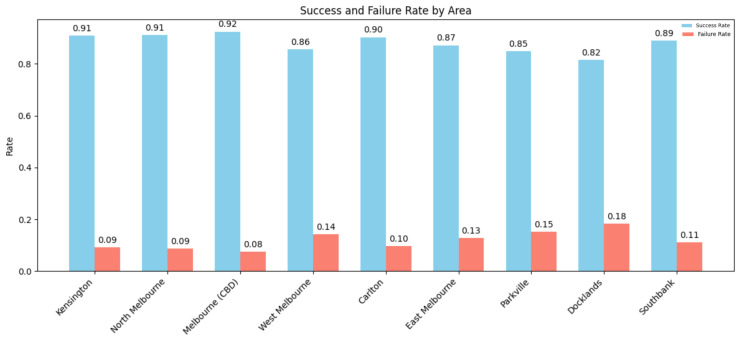
Success and failure rate of daily paring request by zone.

Figure 5 illustrates the overall trends in occupancy and allocation rates by zone, while Figure 6 presents the corresponding success and failure rates. The results confirm that success rates exceed 90% in most zones with high or moderate demand, whereas failure rates are higher in low-demand areas like Docklands and Parkville. This reinforces the capacity of the model to prioritize allocation where needed, and to conserve resources in regions with limited requests.

These patterns demonstrate that the MARL-GCN model effectively adjusts its allocation strategy in response to spatial demand differences, supporting balanced resource distribution across the city.

## 6. Conclusions

Acquiring high-quality spatiotemporal parking demand and supply data remains a significant challenge due to the variability of urban traffic conditions and the limited deployment of parking sensors. The complexities introduced by dynamic demand patterns and spatial dependencies further complicate the accurate prediction and allocation of parking resources. To address these challenges, this paper presents a Multi-Agent Reinforcement Learning (MARL)-based framework, incorporating Graph Convolutional Networks (GCNs) into the MARL-GCN model, designed to optimize on-street parking allocation. The model effectively captures spatial correlations between parking areas and the temporal variations in parking demand, providing a scalable solution for urban parking management.

To account for the diversity in parking demand across different urban areas, we develop a task-specific spatial model to represent the unique spatial relationships between parking spots and demand grids. Additionally, to enhance the allocation process, we introduce a time-adaptive learning module that captures the dynamic, nonlinear relationships between parking supply and demand. This enables the model to effectively address fluctuations in parking demand and allocate spaces in real-time.

Moreover, in response to the challenges of competition and cooperation in parking allocation, the MARL-GCN model incorporates an adaptive framework capable of adjusting its strategy according to peak and off-peak demand variations, ensuring efficient resource allocation throughout the day. The model outperforms traditional strategies, such as first-in first-out (FIFO) and Service in Random Order (SIRO), particularly in high-demand areas, such CBD and Southbank.

The results of this study demonstrate that the MARL-GCN model effectively tackles complex, large-scale parking allocation tasks in dynamic urban environments. However, potential limitations remain. The model does not incorporate parking pricing strategies or behavioral factors such as user preferences and compliance, which are important for fully capturing real-world decision-making dynamics.

Future work may focus on improving the model’s generalizability by integrating real-time traffic and behavioral data, exploring lightweight deployment strategies, and incorporating pricing mechanisms to enhance policy responsiveness and user engagement in broader urban applications.

## Figures and Tables

**Table 1:** Parking requests and available slots by area.

Description Area	Melbourne City	
Clue Area	Number of Parking Slots	Parking Requests per Day
Carlton	71	805
Docklands	17	4191
East Melbourne	37	2283
Kensington	117	1
CBD	83	14,687
North Melbourne	94	2452
Parkville	34	411
Southbank	40	2129
West Melbourne	48	2384
Total	541	29,342

**Table 2:** Model performance comparison with baseline.

	Service Volume	Occupancy Rate (%)	Allocation Rate (%)	Inference Time/Request (s)
MARL-GCN	27,071	66.42	92.24	0.17
FIFO	19,309	65.54	65.81	0.15
SIRO	20,945	57.77	71.40	0.12

**Table 3:** Average daily model performance by zone.

Name of Area	Service Volume	Occupancy Rate (%)	Allocation Rate (%)
Kensington	1	65.42	90.85
North Melbourne	2491	67.31	91.2
Melbourne (CBD)	14,918	68.02	92.45
West Melbourne	2422	64.23	85.67
Carlton	818	66.3	90.3
East Melbourne	2319	63.95	87.18
Parkville	417	64.35	84.8
Docklands	1523	61.29	81.57
Southbank	2163	65.12	88.9

## Data Availability

The datasets used in this research were download from Melbourne open data platform. The dataset regarding on-street parking events in 2019 is available at https://data.melbourne.vic.gov.au/explore/dataset/on-street-car-parking-sensor-data-2019/information/ (accessed on 7 October 2023), the dataset of landmarks and places of interest is available at https://data.melbourne.vic.gov.au/explore/dataset/landmarks-and-places-of-interest-including-schools-theatres-health-services-spor/table/?location=15,-37.82075,144.96226&basemap=mbs-7a7333 (accessed on 29 November 2023), the dataset of the distribution of cafés, restaurants, and bistros is available at https://data.melbourne.vic.gov.au/explore/dataset/cafes-and-restaurants-with-seating-capacity/information/ (accessed on 15 December 2023), the dataset of the location and industry classification of business establishments is available at https://data.melbourne.vic.gov.au/explore/dataset/business-establishments-with-address-and-industry-classification/information/ (accessed on 15 December 2023), and the dataset of residential dwellings is available at https://data.melbourne.vic.gov.au/explore/dataset/residential-dwellings/table/?sort=-dwelling_number (accessed on 15 December 2023).
